# The prognostic effects of circulating myeloid-derived suppressor cells in non-small cell lung cancer: systematic review and meta-analysis

**DOI:** 10.1007/s10238-022-00946-6

**Published:** 2022-11-19

**Authors:** Giuseppe Bronte, Luana Calabrò, Fabiola Olivieri, Antonio Domenico Procopio, Lucio Crinò

**Affiliations:** 1 Department of Clinical and Molecular Sciences (DISCLIMO), Università Politecnica Delle Marche, Via Tronto 10/A, Ancona, Italy; 2 Clinic of Laboratory and Precision Medicine, National Institute of Health and Sciences On Ageing (IRCCS INRCA), Ancona, Italy; 3 Medical Oncology Unit, University Hospital of Ferrara, Ferrara, Italy; 4 Department of Medical Oncology, IRCCS Istituto Romagnolo Per Lo Studio Dei Tumori (IRST) “Dino Amadori”, Meldola, Italy

**Keywords:** Myeloid-derived suppressor cells, Non-small cell lung cancer, Prognosis

## Abstract

Immunotherapy is the main standard treatment for non-small cell lung cancer (NSCLC) patients. Immune suppressive cells in tumor microenvironment can counteract its efficacy. Myeloid-derived suppressor cells (MDSCs) include two major subsets: polymorphonuclear (PMN-MDSCs) and monocytic (M-MDSCs). Many studies explored the prognostic impact of these cell populations in NSCLC patients. The aim of this systematic review is to select studies for a meta-analysis, which compares prognosis between patients with high vs low circulating MDSC levels. We collected hazard ratios (HRs) and relative 95% confidence intervals (CIs) in terms of progression-free survival (PFS) or recurrence-free survival (RFS), and overall survival (OS). Among 139 studies retrieved from literature search, 14 eligible studies (905 NSCLC patients) met inclusion criteria. Low circulating MDSC levels favor a better PFS/RFS (HR = 1.84; 95% CI = 1.28–2.65) and OS (HR = 1.78; 95% CI = 1.29–2.46). The subgroup analysis based on MDSC subtypes (total-, PMN-, and M-MDSCs) obtained a statistical significance only for M-MDSCs, both in terms of PFS/RFS (HR = 2.67; 95% CI = 2.04–3.50) and OS (HR = 2.10; 95% CI = 1.61–2.75). NSCLC patients bearing high M-MDSC levels in peripheral blood experience a worse prognosis than those with low levels, both in terms of PFS/RFS and OS. This finding suggests that detecting and targeting this MDSC subset could help to improve NSCLC treatment efficacy.

**Supplementary Information:**

The online version contains supplementary material available at 10.1007/s10238-022-00946-6.

## Introduction

Non-small cell lung cancer (NSCLC) is one of the malignancies with the worst prognosis. According to Surveillance, Epidemiology, and End Results (SEER) database, between 2011 and 2017 the 5-year relative survival rates for these neoplasms at any SEER stages (localized, regional, or distant) were 26% for NSCLC [1]. The major causes of this extremely poor prognosis can be attributed to the lack of tests for early diagnosis and the lack of significant improvement of treatment efficacy in the advanced setting. However, the recent availability of immune checkpoint inhibitors (ICIs), as an alternative to chemotherapy or in combination with it, could slightly change the survival rates in the next years. Nowadays, ICIs represent the best option for the upfront NSCLC treatment. The combination of pembrolizumab with cisplatin and pemetrexed is recommended for non-squamous non-oncogene-addicted NSCLC [2] and pembrolizumab plus carboplatin and paclitaxel for squamous NSCLC [3].

The efficacy of ICIs can be limited by the presence or activation of suppressive cells. Among these ones myeloid-derived suppressor cells (MDSCs) showed prognostic effects in patients treated with chemotherapy and also in those treated with an ICI as observed recently.

MDSCs belong to a heterogeneous population of immature myeloid cells. These cells can increase during tumor progression as a consequence of chronic inflammation because of soluble or exosome-bound tumor-derived factors. MDSCs include two major subpopulations: polymorphonuclear (PMN-MDSC) characterized by CD11b + CD14-CD15 + or CD11b + CD14-CD66b +  and monocytic (M-MDSC) as CD11b + CD14 + HLA-DR-/lowCD15 cells. CD33 marker for myeloid cells can be considered as an alternative to CD11b. However, these surface markers cannot distinguish monocytes from M-MDSC and neutrophils from PMN-MDSC. Currently, a combination of markers specific for MDSCs has not been known yet [4]. PMN-MDSCs and M-MDSCs have different transcriptomic profiles in comparison with neutrophils and monocytes, respectively. Moreover, some gene expression patterns are more characteristic of PMN-MDSCs than M-MDSCs, e.g., PMN-MDSCs show higher expression of genes related to Janus kinase (JAK) and signal transducer and activator of transcription (STAT) signaling, whereas phosphatidylinositol 3-kinase (PI3K), interleukin 6 (IL-6), and transforming growth factor-beta (TGF-β) were upregulated in M-MDSCs [5].

A significant increase of MDSCs was described in lung cancer, breast cancer, and head and neck cancer. High levels of circulating MDSCs were associated with disease stage and tumor burden [6]. Circulating MDSCs levels in peripheral blood from patients with various malignancies can be tenfold higher than healthy donors, and this favors tumor growth and metastatic spread [7, 8]. MDSCs suppress the immune system inducing a dysfunction of T cells through the production of higher amounts of TGF-beta [9, 10].

The role of MDSCs in NSCLC was studied in some murine models. Among these studies, one investigated B7-H3 + MDSCs in the tumor microenvironment. An association between the increased frequency of these cells and lung tumor progression was observed [11]. In another study on lung cancer-bearing mice, cisplatin treatment elicited a MDSCs increase, and this treatment-related change was mediated by galectin-3 [12]. The effect of some carcinogens on MDSCs was explored. The use of carbon nanotubes in the lungs of mice stimulated the recruitment and accumulation of MDSCs and their relative production of TGF-β, with consequent increase of the tumor burden [13]. Similarly, the exposition of mice to cigarette smoke favored the accumulation of MDSCs in many organs, but these cells were not immunosuppressive, and changed to immunosuppressive phenotype when urethane was added to cigarette smoke [14]. The immunosuppressive activity is exerted through arginases, nitric oxide, reactive oxygen species, but also various cytokines and PD-1/PD-L1 axis. This implies the potential effects of MDSC function on anti-PD-1 and anti-PD-L1 used in NSCLC patients [15].

In a meta-analysis by Wang et al., higher pretreatment circulating MDSC levels showed a potential prognostic role in patients with solid tumors. However, they included all cancer types, irrespective of cancer stage. This meta-analysis was performed in 2018, and it included just 3 studies on 259 NSCLC patients [16]. Given that further studies explored MDSCs in NSCLC in the last years, a specific systematic review, and potential relative meta-analysis, is needed to clarify the prognostic impact of circulating MDSCs in patients with this malignancy. MDSCs are gaining relevance as biomarker of resistance to cancer treatments, and new therapeutic strategies targeting these cells are under development.

MDSCs can also contribute to the pathogenesis of hematologic malignancies through the suppression of the immune response against malignant cells. Besides, MDSCs may be part of the malignant clone, such as in myelodysplasia [17]. Some treatment strategies applied in hematologic malignancies can influence the MDSC amount and function [18]. Among these, all-trans retinoic acid induces MDSC differentiation with a consequent reduction of immune-suppressive MDSCs [19]. Moreover, Daratumumab, an anti-CD38 monoclonal antibody delivered in multiple myeloma patients to eradicate plasma cells, may also contribute to the elimination of CD38-expressing MDSCs [20]. Other treatment strategies are under investigation to reduce immunosuppressive MDSCs. These include gemtuzumab ozogamicin, an anti-CD33 antibody conjugated to a cytotoxic agent [21].

In this systematic review, we gathered and analyzed together the findings about the prognostic role of MDSCs in NSCLC patients.

## Methods

### Review design and registration

This work is designed as a systematic review of studies including prognostic evaluations. This is the question to address: Are high levels of circulating MDSCs a prognostic indicator in NSCLC patients? We used the PICOTS system (Population, Index prognostic factor, Comparison, Outcome, Timing, Setting) [22]. The population include patients with a diagnosis of NSCLC; the index prognostic factor is MDSCs level in peripheral blood; the comparison is circulating MDSCs high vs low levels as defined by the various cutoff values identified for each study; the outcome measures are the hazard ratios for overall survival (OS) and progression-free survival (PFS), or disease-free survival (DFS), or recurrence-free survival (RFS); the timing is referred to pretreatment blood withdrawal; all settings of treatment for NSCLC patients were included.

The systematic review protocol was registered on the International Prospective Register of Systematic Reviews (PROSPERO) (registration code: CRD42022331407).

### Search strategy

The search for publications on this topic was performed in Medline, by using PubMed online service, updated in April 2022. The following search terms combination was used: *(MDSC OR "myeloid derived suppressor cells") AND (NSCLC OR "non small cell lung cancer")*. We limited the search to studies in the English language and in the period since the year 2000. The results were supplemented with manual searches of meeting proceedings and references from already known selected articles. We did not find a similar systematic review on this topic.

### Study selection

To be included the records from database searching or other sources needed to 1) focus on MDSCs; 2) be original researches; 3) apply to a clinical setting; 4) include patients with a diagnosis of NSCLC; 5) include patients who were evaluated for circulating MDSCs in the peripheral blood; 6) have performed a prognostic evaluation with the association between MDSC levels (stratified as high, if above a cutoff value, or low, if below a cutoff value) and survival providing a hazard ratio (HR) with a 95% confidence interval (CI) for survival outcomes, or enough data to calculate these measures. The records that met the above criteria were deeply analyzed in the full text articles. These articles were excluded if they (1) lacked information needed for the analysis; (2) combined MDSC assessment with other cell subtypes; (3) focused on MDSCs in tumor microenvironment; (4) blood withdrawal was performed after starting a cancer treatment; (5) if data from NSCLC patients were analyzed together with those from patients bearing other tumor types.

### Data extraction

We extracted data from each eligible study and reported these into a spreadsheet. The data we considered included the name of the first author, year of publication, country, NSCLC histology (i.e., squamous, non-squamous, mixed, unknown), clinical or pathological NSCLC stage (i.e., I to IV), type of treatment (i.e., chemotherapy, immunotherapy, chemo-immunotherapy, target therapy, radiotherapy, chemoradiotherapy, surgery, other, unknown), sample size, patient’s age (median and/or range), MDSC subtypes (i.e., total-MDSCs, PMN-MDSCs, M-MDSCs, both), MDSC markers, way to define the cutoff value for MDSCs, cutoff value, HRs with relative 95% CIs. HRs and 95% CIs were extracted for OS and PFS/RFS/DFS. If HRs and relative 95% CIs were not reported in the article, we estimated these measures from the Kaplan–Meier curves, according to the methodology that was previously described [23, 24]. Potential disagreements were solved by a consensus between the authors.

### Statistical analysis

The outcomes selected for meta-analysis comparisons were OS and PFS/RFS/DFS stratified according to high or low values of circulating total-MDSCs, PMN-MDSCs, or M-MDSCs. Once we collected the HRs and relative 95% CIs from the articles or estimated these measures through the specific methodology, we used these data to calculate the pooled HRs and 95% CIs for each outcome. Study-specific HRs were weighted according to the Mantel–Haenszel method. If HR was > 1, the patients with low MDSCs value would have a better outcome, and vice versa.

Heterogeneity was evaluated through the *χ*^*2*^ test and expressed as the I^2^ index (25% = low, 50% = medium, 75% = high) [25]. If heterogeneity was observed, we used a random effects model. Otherwise, a fixed effects model was used in the meta-analysis. Publication bias was evaluated by a visual representation as a Begg’s funnel plot and also calculated through Egger’s test [26, 27]. In case of publication bias, Duval and Tweedie’s trim-and-fill method was used to calculate an adjusted effect size for HRs [28]. *P* values < 0.05 were considered statistically significant. We carried out statistical analyses by using Review Manager (RevMan, version 5.3) and Comprehensive Meta-Analysis software (CMA, version 3.3).

We drafted this manuscript following the Preferred Reporting Items for Systematic Reviews and Meta-Analyses (PRISMA) guidelines (Supplementary Table 1).

## Results

### Literature search and study selection

**Fig. 1:**
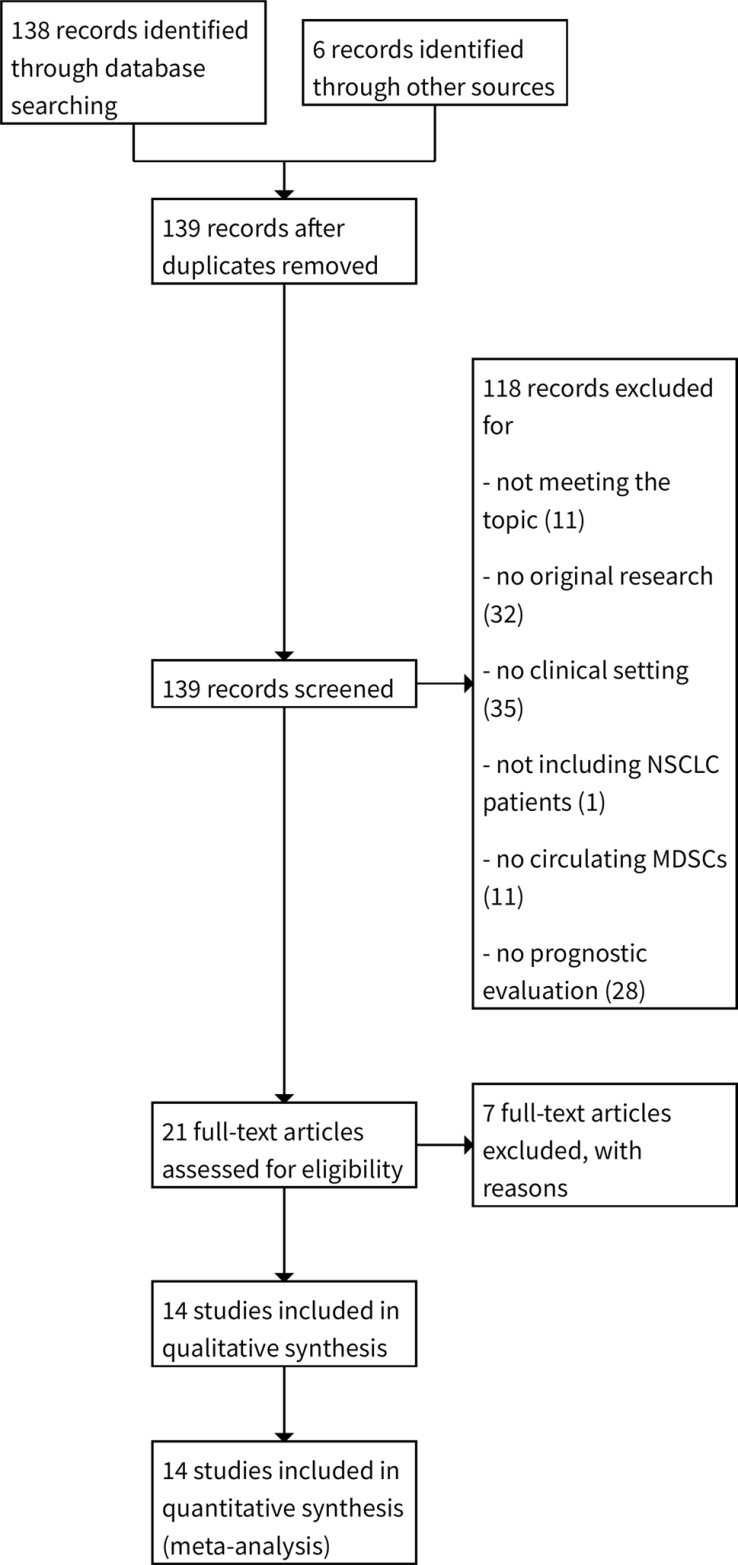
PRISMA flow diagram. The diagram shows the selection of retrieved papers, by using the eligibility criteria. *NSCLC* non-small cell lung cancer, *MDSC* myeloid-derived suppressor cells

The search for articles retrieved 138 records from databases and 6 through manual search. After duplicate removal, 139 records underwent evaluation for study selection. The screening of the title and abstract of the records led to exclude 118 of these: (a) 11 not meeting the topic of this review; (b) 32 not being original research articles; c) 35 not regarding a clinical setting; (d) 1 not including NSCLC patients; (e) 11 not studying circulating MDSCs; (f) 28 not performing prognostic evaluation. Then, we examined the full text of the remaining 21 articles for eligibility. Among these articles, we further excluded 7 of them because met the above-described exclusion criteria. We obtained 14 eligible articles for qualitative and quantitative analysis [29–42]. The selection PRISMA diagram is depicted in Fig. 1.

### Study characteristics

The selected 14 studies included 905 NSCLC patients. Nine studies were performed in Western countries and 5 in Eastern countries. The majority of the studies (n = 9) included both non-squamous and squamous NSCLC histology, mainly in the advanced stages (III-IV). Three studies evaluated patients undergoing immunotherapy, and 6 included those experiencing a cytotoxic treatment. The median age ranges between 46 and 70 years. Two studies evaluated circulating MDSCs as a total entity, 2 detected only PMN-MDSCs, 6 only M-MDSCs, 4 detected both subtypes but distinguishing them. The majority of the eligible studies (n = 8) used the median to define the cutoff value. The cutoff values ranged between 1 and 20%, in one study the cutoff value was expressed as number of cells per microliter, and 3 articles did not report the cutoff value.

Two studies analyzed only PFS, 1 only RFS, 4 only OS; a half of the study explored both PFS and OS. Table 1 encloses the detailed study characteristics.

**Table 1:** Main characteristics of 14 eligible studies included in the meta-analysis

Author, year	Country	Histology	Stage	Treatment	Sample size	Age	MDSC type	MDSC markers	MDSC cutoff method	Cutoff value	Outcome
Bronte, (2022)	Italy	Non-squamous	III-IV	Immunotherapy	22	70.1 (64.8–75.0)	M-MDSCs	CD14 + HLA-DR − /lowCD11b + CD33 +	Median	1.9%	PFS, OS
Zahran, (2021)	Egypt	Mixed	III-IV	Chemotherapy	40	58.5 ± 1.7	M-MDSCs	CD14 + HLA-DR-	ROC curve	13%	OS
Koh (2020)	Korea	Mixed	I-IV	Immunotherapy	132	62 (34–88)	Both	PMN-MDSCs: Lin − CD15 + CD14 − CD11b + HLA-DR − /low; M-MDSCs: Lin − CD15 − CD14 + HLA-DR − /low	Median	NR	PFS, OS
Passaro, (2020)	Italy	Mixed	III-IV	Immunotherapy	53	64 (56–70)	PMN-MDSCs	SSClow Lin − /HLA-DR − /LOW CD33 + /CD13 + /CD11b + /CD15 + /CD14 −	Other	6 cell/μL	PFS, OS
Li, (2019)	USA	Mixed	IV	Unknown	34	66 (38–84)	Total-MDSCs	CD33 + /CD11b + /HLA-DRlow	Median	18%	PFS, OS
Riemann, (2019)	Germany	Mixed	I-III	Surgery	58	67 ± 9	M-MDSCs	HLA-DRlow	Other	3%	OS
Yamauchi, (2018)	Germany	Mixed	I-III	Surgery	42	63 (48–79)	Both	M-MDSCs: HLA-DR-/lowCD11b + CD14 + CD15-PMN-MDSCs: HLADR-/lowCD11b + CD14-CD15 +	Other	NR	RFS
Barrera, (201)8	Mexico	Unknown	III-IV	Chemotherapy	90	NR	PMN-MDSCs	CD66b + CD11b + CD15 + CD14 −	Other	8%	PFS, OS
Feng, (2018)	China	Non-squamous	IV	Target therapy	55	BEV + TKI: 62 ± 10.7(42–84) TKI: 68 ± 11.6(53–89)	M-MDSCs	CD11b + CD14 + S100A9 +	Median	16,3%	PFS, OS
Hansen, (2015)	Norway	Unknown	III	Chemoradiotherapy	23	59.5 (46–78)	Total-MDSCs	Lin − /loHLA‑DR − CD33 + CD11b +	Median	1%	OS
de Goeje, (2015)	The Netherlands	Non-squamous	IV	Chemotherapy	105	61 ± 8.3	Both	PMN-MDSCs	CD11b + CD14-HLA-DR-CD33 + CD15 + M-MDSCs: CD11b + CD14 + HLA-DR-CD33 + CD15 +	Median	NR, OS
Vetsika, (2014)	Greece	Mixed	III-IV	Unknown	110	68 (53–89)	Both	M-MDSCs: HLA-DR-Lin-CD33 + CD11b + CD14-CD15 ± PMN-MDSCs: HLA-DR-Lin-CD33 + CD11b + CD14 + CD15-	Other	2,2%	PFS, OS
Huang, (2013)	China	Mixed	III-IV	Chemotherapy	89	46.78 ± 9.60	M-MDSCs	CD14 + HLA-DR-/low	Median	9,43%	PFS
Feng, (2012)	Taiwan	Mixed	III-IV	Chemotherapy	52	57.6 ± 12	M-MDSCs	CD11b + CD14 + S100A9 +	Median	20%	PFS

*USA* United States of America;* MDSCs* myeloid-derived suppressor cells;* M* monocytic;* PMN* polymorphonuclear;* CD* cluster of differentiation;* HLA-DR* human leucocyte antigen DR;* Lin* leukocyte lineage;* PFS* progression-free survival;* OS* overall survival;* RFS* recurrence-free survival

A meta-analysis of these studies was performed to achieve pooled HRs for OS and PFS/RFS. Six articles reported HRs and relative 95% CIs. We applied to the remaining 8 studies the previously described methodology for extracting HRs and 95% CIs from Kaplan–Meier.

### Survival analyses

**Fig. 2:**
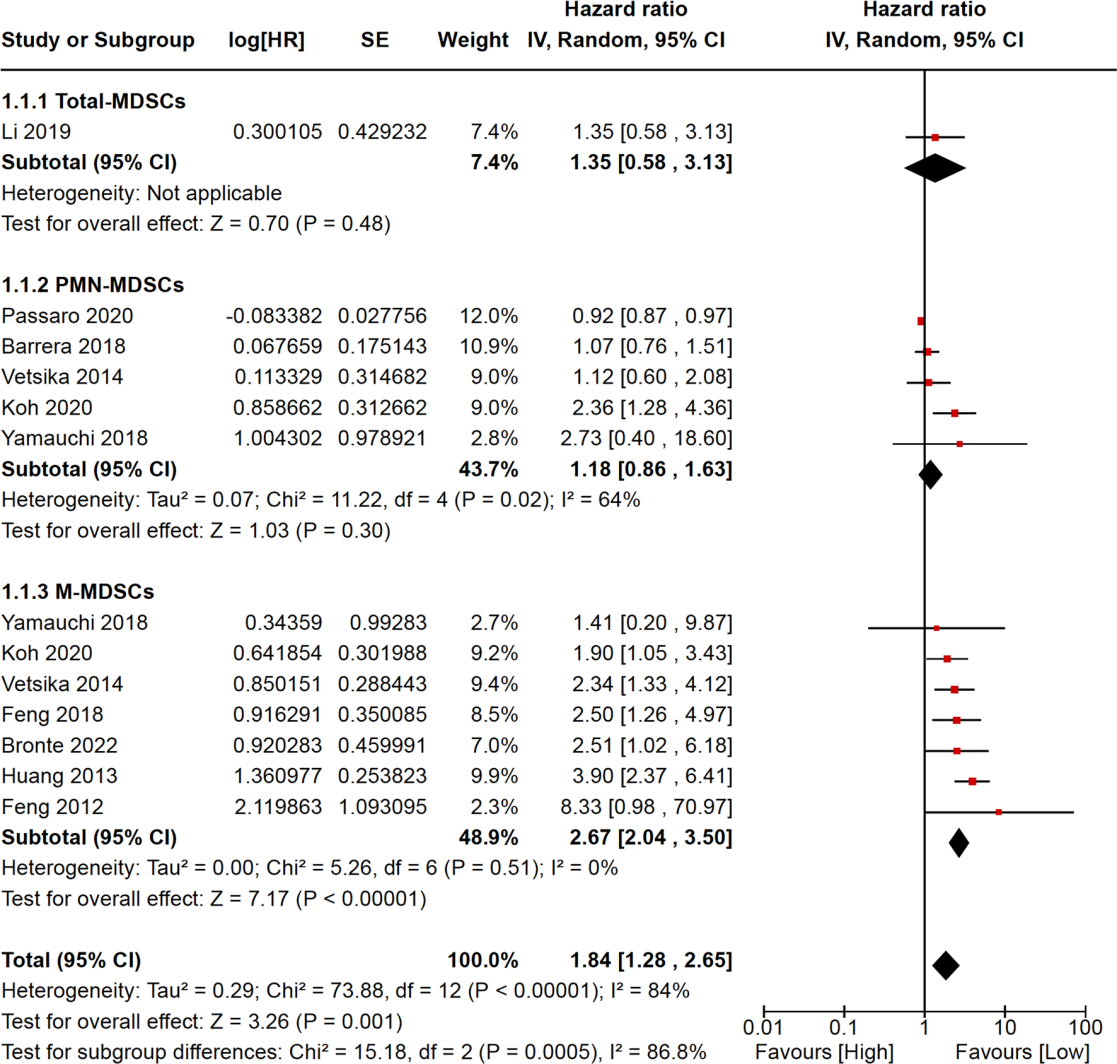
Meta-analysis results for progression-free/relapse-free survival are reported as forest plot, including logarithm of hazard ratio, standard error, weight, hazard ratio with relative 95% confidence intervals for each study. Weight and hazard ratio with relative 95% confidence intervals are reported for pooled analyses (subtotals and total). *MDSCs* myeloid-derived suppressive cells, *PMN* polymorphonuclear, *M* monocytic, *log[HR]* logarithm of hazard ratio, *SE* standard error, *IV* instrumental variables, *CI* confidence intervals, *df* degrees of freedom, *P* p-value, *I2* heterogeneity statistic I2, *Z* z-test

Ten studies including 679 NSCLC patients were analyzed for PFS/RFS. Of these, 1 evaluated total-MDSCs, 5 PMN-MDSCs, and 7 M-MDSCs. The pooled HR for PFS/RFS highlighted that low circulating MDSC levels favor a better survival free of progression/recurrence (HR = 1.84; 95% CI = 1.28–2.65). However, in the subgroup analysis according to MDSC subtypes, the statistical significance was achieved for M-MDSCs only (HR = 2.67; 95% CI = 2.04–3.50). The pooled HR for PFS/RFS was calculated through the random effect model, because of significant heterogeneity with the fixed effect model (*p* < 0.00001) (Fig. 2).

**Fig. 3:**
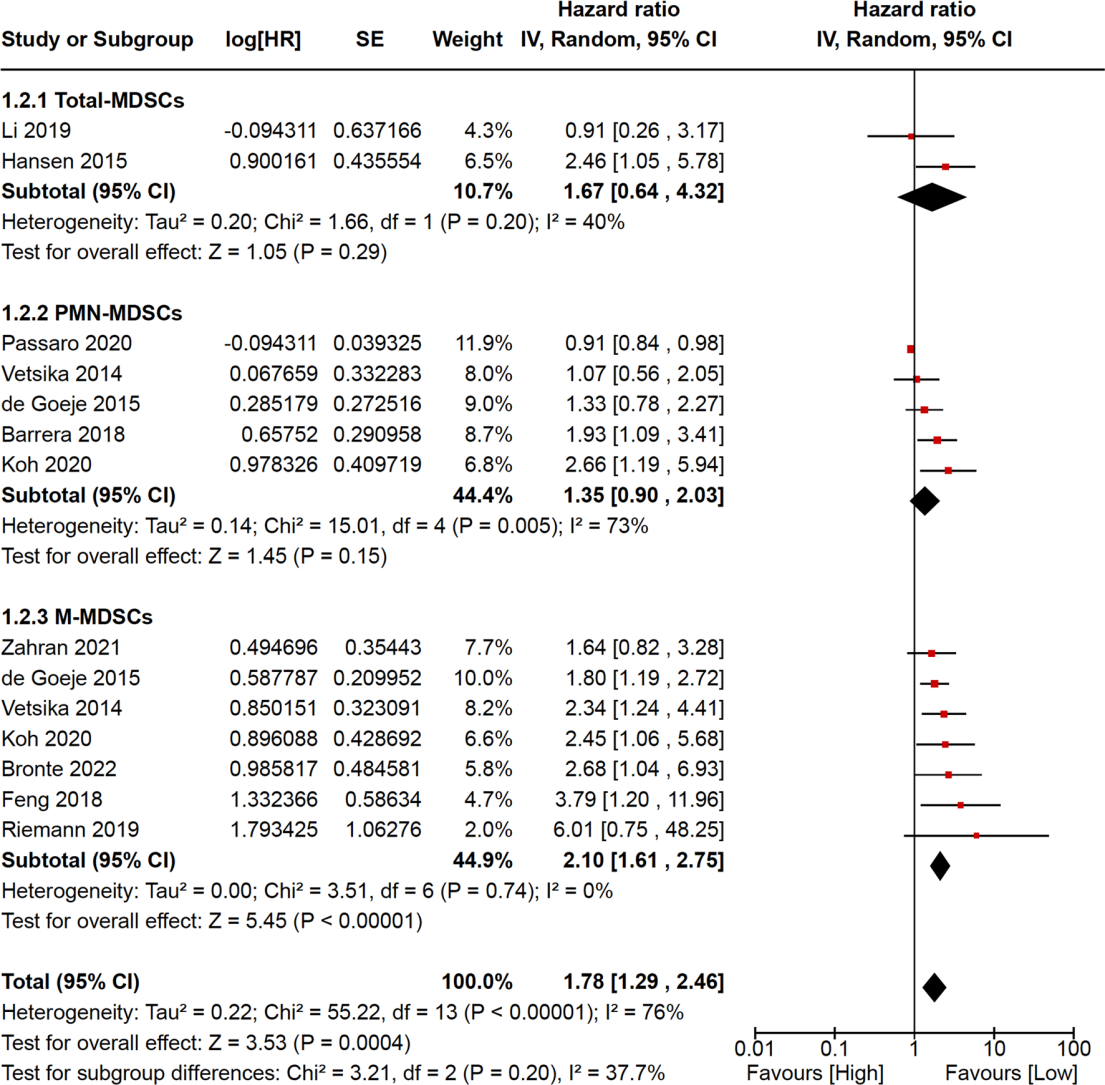
Meta-analysis results for overall survival are reported as forest plot, including logarithm of hazard ratio, standard error, weight, hazard ratio with relative 95% confidence intervals for each study. Weight and hazard ratio with relative 95% confidence intervals are reported for pooled analyses (subtotals and total). *MDSCs* myeloid-derived suppressive cells, *PMN* polymorphonuclear, *M* monocytic, *log[HR]* logarithm of hazard ratio, *SE* standard error, *IV* instrumental variables, *CI* confidence intervals; *df* degrees of freedom, *P* p-value, *I2* heterogeneity statistic I2, *Z* *z*-test

Eleven studies including 722 NSCLC patients were analyzed for OS. Of these, 2 evaluated total-MDSCs, 5 PMN-MDSCs, and 7 M-MDSCs. Similarly to PFS/RFS, the pooled HR for OS shows a better outcome for the patients with low circulating MDSC levels (HR = 1.78; 95% CI = 1.29–2.46). Also in this case, the subgroup analysis according to MDSC subtypes achieved a statistical significance only for M-MDSCs (HR = 2.10; 95% CI = 1.61–2.75). The pooled HR for OS was calculated through the random effect model, because of significant heterogeneity with the fixed effect model (*p* < 0.00001) (Fig. 3).

### Publication bias

**Fig. 4:**
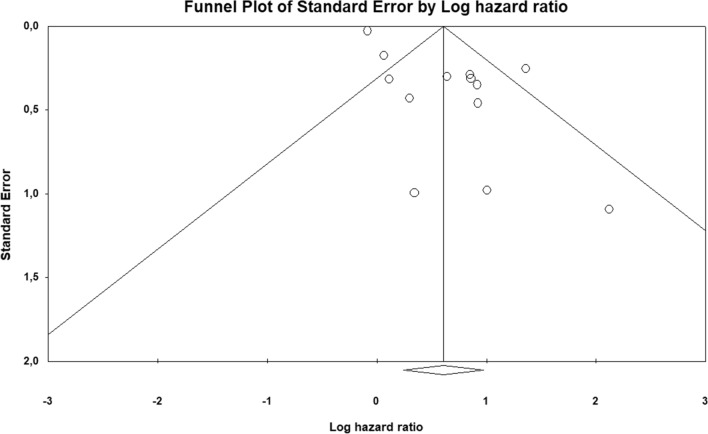
Publication bias for progression-free/relapse-free survival analysis is represented by means of funnel plot, reporting in x-axis the logarithm of hazard ratio and in the y-axis the standard error

**Fig. 5:**
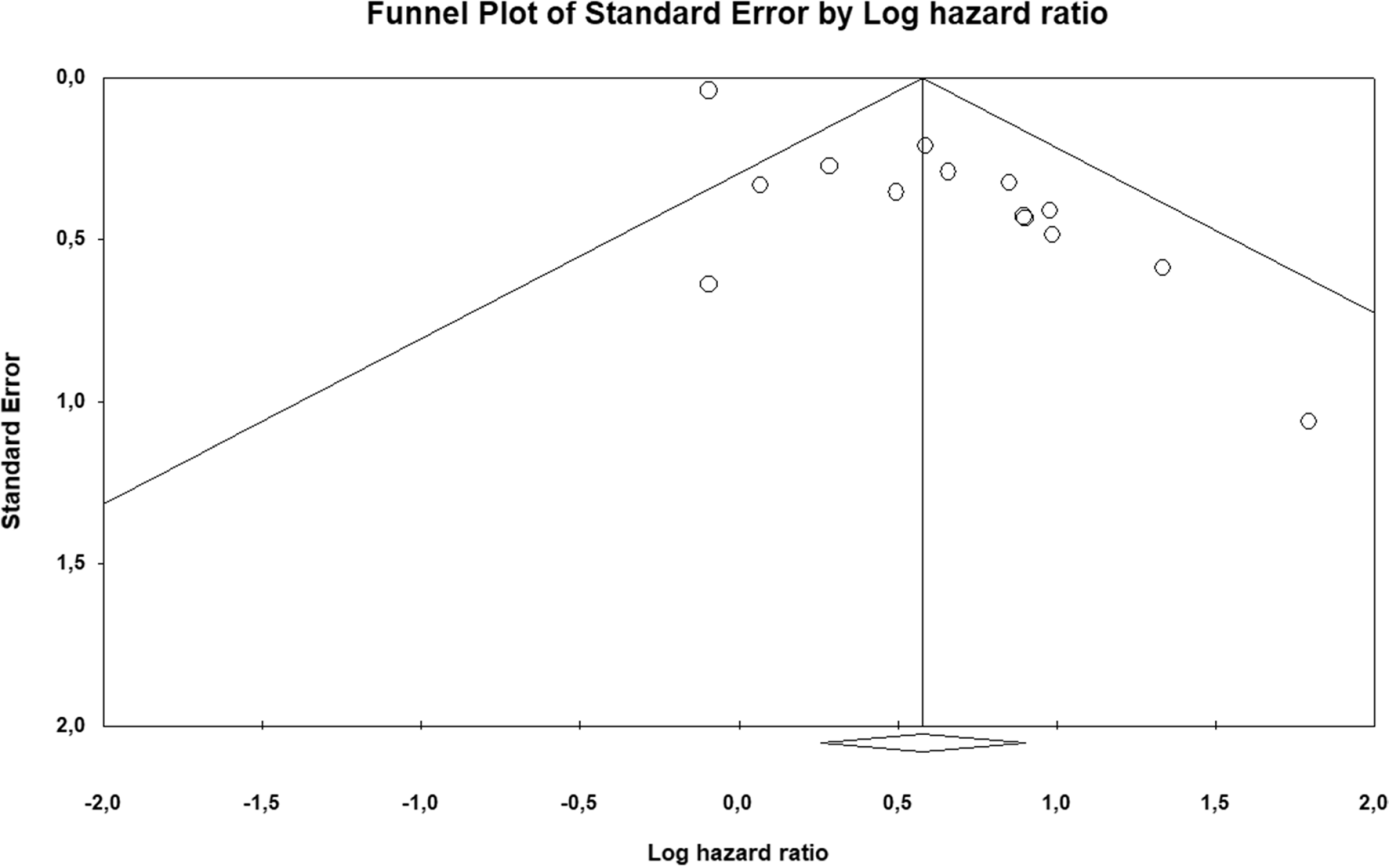
Publication bias for overall survival analysis is represented by means of funnel plot, reporting in x-axis the logarithm of hazard ratio and in the y-axis the standard error

The funnel plot highlights publication bias for the studies included in both the PFS/RFS and OS analyses (Fig. 4 and Fig. 5). This observation is confirmed through the Egger’s tests (*p* = 0.00081 for PFS/RFS; *p* = 0.00002 for OS). To solve this problem we applied the trim-and-fill method, which showed a pooled adjusted HR = 1.51 (95% CI = 1.11–2.08) for PFS/RFS and 1.67 (95% CI = 1.23–2.26) for OS. These adjusted pooled HRs confirmed the statistically significant association between MDSCs low levels and better prognosis.

## Discussion

Our systematic review allowed selecting 14 studies (905 NSCLC patients) for meta-analysis. Unlike a previous meta-analysis about the prognostic role of circulating MDSCs including all solid tumors [16], this study includes more updated studies evaluating the same topic, but specifically in NSCLC patients. The meta-analysis resulted in better survival outcomes in patients with low circulating MDSCs levels, and accordingly, high values can predict a worse prognosis both in terms of PFS/RFS and OS.

The subgroups analysis according to MDSC subtypes (total-, PMN-, and M-MDSCs) highlighted that only M-MDSCs achieve statistically significant prognostic effects. The evidence of this difference between PMN- and M-MDSCs leads to seek the reason. So, an insight in the mechanisms of immunosuppression by MDSCs should be taken into account. MDSCs widely suppress the immune system through various ways: (a) T cell function inhibition through the depletion of amino acids essential for T-cell proliferation; (b) direct inhibition of T cell viability and migration through PD-1/PD-L1 signaling pathway; (c) T cell apoptosis through the production of nitric oxide (NO) and reactive oxygen species (ROS); (d) CD4 + T cell transformation into regulatory T cells (Tregs) through TGF-β; (e) macrophage repolarization toward M2 phenotype; (f) natural killer (NK) cell alteration through direct cell–cell contact with IFN-γ reduction [43–46]. However, different levels of T-cell suppression by these two subsets of MDSCs are known: PMN-MDSCs induce antigen-specific T-cell tolerance through high levels of ROS and low levels of NO [47, 48]. Conversely, M-MDSCs impair both antigen-specific and neoantigen-specific T-cell responses through low ROS levels and high NO levels, and this inhibition appears to be continuous [49]. Given that just M-MDSCs can interfere with neoantigen-specific immune activity, NSCLC can be particularly affected by this interference [50, 51]. Aside from these assumptions, studies specifically addressing this question are needed. This address becomes relevant both for the detection of immunotherapy resistance biomarkers and for the definition of therapeutic strategies targeting MDSCs.

We are aware this meta-analysis holds some limitations. (1) Various markers characterized MDSC subtypes across the analyzed studies. So, we trusted on the MDSC classification established by the authors for each study. The design for future studies on MDSCs should refer to previously published recommendations [4]. However, HLA-DR and CD14 are the most common markers used for this characterization as Table 1 highlights. (2) HRs and relative 95% CIs were calculated from Kaplan–Meier survival curves for more than a half of included studies, because the authors did not report these measures in the paper. This matter suggests that future studies on the prognostic evaluation of MDSCs should always report HRs and 95% CIs to be taken into account for publication. (3) In this meta-analysis we obtained heterogeneity, which can be a consequence of study design and different populations. We sought to minimize this problem applying the random effect model. (4) Publication bias emerged through qualitative analysis of funnel plots and quantitative results of Egger’s test. We solved this matter with trim-and-fill method, but likely pooled adjusted survival measures did not change. (5) We excluded the studies on intratumoral MDSCs focusing on MDSCs from peripheral blood. This choice is based on the assumption that MDSCs are produced in the bone marrow, first migrate to the peripheral blood, and then reach tumor microenvironment. This selection guaranteed not to confuse different approaches, but MDSCs from the tumor microenvironment can also influence the prognosis. Future studies should evaluate concurrently intratumoral and circulating MDSCs and possibly compare their prognostic effects.

## Conclusions

The results of this meta-analysis suggest that NSCLC patients with high levels of circulating M-MDSCs undergo a higher risk of recurrence/progression and death than those with low levels. PMN-MDSCs did not achieve the same prognostic effects. These results should be taken into account for the development of new treatment strategies in NSCLC patients, so that M-MDSCs could become the real target. From the limitations of this meta-analysis we can learn that for future studies we have to consider recommendations for the choice of MDSC markers, HRs and 95% CIs should always be reported in the paper, and MDSCs in tumor tissue should be studied concurrently with circulating MDSCs.

## Supplementary Information

Below is the link to the electronic supplementary material.

Supplementary file1 (DOCX 35 kb)

## Data Availability

Details on extracted data are available by the corresponding author upon reasonable request.
